# Lifestyle and socioeconomic determinants of diabetes: Evidence from country-level data

**DOI:** 10.1371/journal.pone.0270476

**Published:** 2022-07-28

**Authors:** Selena E. Richards, Chandana Wijeweera, Albert Wijeweera

**Affiliations:** 1 Department of Chemistry, Khalifa University of Science and Technology, Abu Dhabi, United Arab Emirates; 2 Center for Biotechnology (BTC), Khalifa University of Science and Technology, Abu Dhabi, United Arab Emirates; 3 Faculty of Medicine, Health and Human Sciences, Macquarie University, Sydney, Australia; 4 Department of Humanities and Social Sciences, Khalifa University of Science and Technology, Abu Dhabi, United Arab Emirates; PLOS ONE, UNITED KINGDOM

## Abstract

**Objective:**

The objectives of the study is to investigate the global socioeconomic risk factors associated with diabetes prevalence using evidence from available country-level data.

**Design:**

A cross-sectional study based on (2010 & 2019) countrywide Health Nutrition and Population Statistics data.

**Population:**

People ages 20–79 who have diabetes.

**Setting:**

One hundred and thirty-two countries or territories in the world.

**Primary outcome measure:**

Diabetes prevalence rates were determined from (2010 & 2019) countrywide Health Nutrition and Population Statistics (Health Stats, World Bank Group).

**Results:**

In 2010, a 1% increase in per capita income and total tobacco consumption is associated with a 0.92% (95% CI 0.64% to 1.19%) and 0.02% (95% CI 0.006% to 0.047%) increase in diabetes prevalence respectively; and a 1% increase in alcohol consumption is associated with a -0.85% (95% CI -1.17% to -0.53%) decrease in diabetes prevalence. Statistically significant socioeconomic and lifestyle indices positively associated with diabetes prevalence included gross national income; overweight prevalence (BMI>25 kg/m^2^); and tobacco consumption. Statistically significant inverse associations with global diabetes prevalence included total population size; unemployment and alcohol consumption. The 2019 data was removed due to sparsity of data.

**Conclusion:**

Statistically significant global lifestyle and socioeconomic determinants of diabetes prevalence include alcohol consumption; tobacco consumption; overweight prevalence; per capita income; total population and unemployment rates. Determinants of diabetes include modifiable risk factors which are consistent at both the micro and macro level and include tobacco consumption and overweight prevalence. Factors which are non-modifiable and warrant further investigation include total population and unemployment rates, which were inversely associated with diabetes prevalence and are a product of other underlying factors. Other determinants such as alcohol consumption was also inversely associated with diabetes prevalence, but has been observed to have both negative and positive associations with diabetes at the micro-level. These associations were dependent upon the amount of alcohol consumed. Global cut-off point of alcohol consumption is critical to establish global policies to reduce diabetes prevalence. Overall, the use of cross-sectional based study for country level aggregate data is a critical tool that should be considered when making global joint strategies or policies against diabetes in both data analysis and decision making.

## 1. Introduction

Diabetes has been conventionally perceived as a “disease of excess”, affecting primarily older populations in developed countries. Statistics show this is no longer the case with diabetes now being prevalent in all populations and pervasive through all strata of society [1, 2].

In 2019, the latest global data from the International Diabetes Federation (IDF) estimated a prevalence of 463 million people currently living with diabetes [2]. This is an alarming rise from the 151 million people that were estimated to live with the condition when the IDF first published global prevalence data in 2000 [2]. Current models project this number to increase to more than 700 million by 2045 [2]. The percentage distribution of the burden of disease of diabetes is currently higher in urban (10.8%) than rural (7.2%) areas, and more prevalent in high-income (10.4%) than low-income (4.0%) countries [2]. However, this representation of the data is misleading since the populations of low- and middle-income countries are on average much higher than those of developed countries [2]. In fact, when examined as raw data, 4 out of every 5 patients with diabetes currently reside in a low or middle-income country [2]. This statistic is likely to worsen over time with a projected rise in diabetes in Africa (143%), Middle East and Northern Africa (96%), Southeast Asia (74%), and South and Central America (55%) by 2045 [2].

Based on statistics from the IDF, there were more than 4.2 million diabetes-related deaths in 2019 [2, 3]. This is in contrast to COVID-19 which was responsible for the same number of deaths over 20 months from its inception (November 2019 –July 2021) [3]. However, it is important to note that these numbers might overlap because of the possibility that patients with diabetes who died of Covid-19 could be included in both categories. For example, a recent study based on a data set from England showed that Covid-19 comorbidities such as Type 2 diabetes could significantly affect the severity of the disease [4]. Nonetheless, it is evident that diabetes has reached the proportions of an epidemic and as such, should be given the appropriate public health priority [3]. Traditional public health approaches to disease control are targeted at communicable diseases and include active surveillance, risk factor identification, and reduction strategies, case identification, and monitoring outcomes [5]. It is difficult to directly translate such a strategy to a non-communicable disease such as diabetes however, it is well understood that primary prevention strategies for diabetes should be aimed at modifying underlying determinants of health [6]. Numerous studies have attempted to uncover socioeconomic and lifestyle-based risk factors affecting the prevalence of diabetes, however, the majority of these have used survey-based, micro-level data from hospitals, cities, or regions of a country [7–16]. Hence, statistical inferences cannot be reliably generalized to the rest of the world.

Despite the global significance of diabetes as a public health issue, very few studies have utilized country-level aggregate data to understand the determinants of diabetes prevalence [1]. The relative absence of macro-level research on this topic has so far been attributed to the paucity of data in this area which has led to a glaring gap in the existing literature [1]. Fortunately, the World Bank has recently published country-level data for the years 2010 and 2019 encompassing more than 250 countries and territories [17]. This data has become available at a crucial time when governments and policymakers need more comprehensive research to better understand the modifiable and non-modifiable risk factors for incident diabetes to underpin robust public health policy. We expect to address the current research gap by performing a cross-sectional regression study of recently published macro-data from the World Bank’s Health Nutrition and Population database to examine country-level evidence on several socioeconomic and lifestyle risks for incident diabetes [17]. This study aims to identify the statistically significant associations between the rising prevalence of diabetes and well-known risk factors of diabetes (such as low SES, high BMI, tobacco use and alcohol consumption). The key global lifestyle and socioeconomic determinates are evaluated and discussed within the context of the current literature.

## 2. Methodology

Publicly available data (2010 & 2019) from Health Nutrition and Population Statistics, World Bank [17] were used to investigate the relationship of key country-level lifestyle and socioeconomic determinates associated with the prevalence of diabetes.

### Data

Lifestyle and socioeconomic determinants of diabetes prevalence were determined utilizing publicly available countrywide Health Nutrition and Population Statistics [17]. The dataset includes annual country-level data on diabetes prevalence; health and socioeconomic factors as well as other variables which may be of interest. The data sources of the datasets include, International Diabetes Federation, Diabetes Atlas; World Health Organization, Global Health Observatory Data Repository; World Bank national accounts data; OECD National Accounts data files; United Nations Population Division. World Population Prospects: 2019 Revision; Census reports and other statistical publications from national statistical offices; Eurostat: Demographic Statistics, United Nations Statistical Division; Population and Vital Statistics Report; U.S. Census Bureau: International Database, Secretariat of the Pacific Community: Statistics and Demography Programme; International Labour Organization and ILOSTAT database.

### Statistical analysis

Global diabetes prevalence data was available for 2010 for 229 countries and territories, 2019 data was omitted due to scarcity of data [17]. Existing literature has identified over 20 potential lifestyle and socioeconomic variables as explanatory variables. Variables that have been used in the literature are: income, prevalence of aggregate tobacco use, prevalence of male tobacco use, prevalence of female tobacco use, prevalence of overweight, total alcohol consumption per capita, male alcohol consumption per capita, female alcohol consumption per capita, unemployment rate, total population, body mass index, physical activity indicator, education level, occupation, nutrition indicator, racial differences, age structure, life expectancy, literacy rate, and gender. However, only nine of those socioeconomic variables were available in the countrywide Health Nutrition and Population Statistics [17]. Multivariate linear regression (MLR) models for the prediction of global diabetes prevalence were estimated from lifestyle and socioeconomic determinants for 132 countries, 97 countries and territories were excluded because the complete set of selected lifestyle and socioeconomic variables were not available for those countries (Table 1). The MLR models was optimized through minimizing multicollinearity and heteroscedasticity.

**Table 1:** Lifestyle and socioeconomic variables used in MLR models. Data obtained from countrywide Health Nutrition and Population Statistics data.

Variables	Description
**DIAB**	Diabetes prevalence (% of population ages 20 to 79). Diabetes prevalence refers to the percentage of people ages 20–79 who have type 1 or type 2 diabetes
**ALCHO**	Total alcohol consumption per capita (litres of pure alcohol, projected estimates, 15+ years of age). Total alcohol per capita consumption is defined as the total (sum of recorded and unrecorded alcohol) amount of alcohol consumed per person (15 years of age or older) over a calendar year, in litres of pure alcohol, adjusted for tourist consumption.
**GNI**	GNI per capita is the gross national income, converted to U.S. dollars divided by the midyear population
**LIEX**	Life expectancy at birth indicates the number of years a newborn infant would live if prevailing patterns of mortality at the time of its birth were to stay the same throughout its life.
**OWEI**	Prevalence of overweight percentage of adults. Prevalence of overweight adults is the percentage of adults ages 18 and over whose Body Mass Index (BMI) is more than 25 kg/m^2^
**POP**	Total population is based on the de facto definition of population, which counts all residents regardless of legal status or citizenship. The values shown are midyear estimates and expressed in millions
**TOBFE**	Prevalence of current tobacco use percentage of female adults. The percentage of the population ages 15 years and over who currently use any tobacco product (smoked and/or smokeless tobacco) on a daily or non-daily basis
**TOBMA**	Prevalence of current tobacco use percentage of male adults. The percentage of the population ages 15 years and over who currently use any tobacco product (smoked and/or smokeless tobacco) on a daily or non-daily basis
**TOBT**	Prevalence of current tobacco use percentage of adults. The percentage of the population ages 15 years and over who currently use any tobacco product (smoked and/or smokeless tobacco) on a daily or non-daily basis
**UNEM**	Unemployment is the percentage of the total labour force (modelled ILO estimate). Unemployment refers to the share of the labour force that is without work but available for and seeking employment.

Summary statistics (mean, variance, and standardized third and fourth moments of a distribution; skewness and kurtosis statistics) were calculated for model variables. Population (POP) data; per capita income (GNI) and alcohol intake (ALCHO) were log-transformed to correct for non-normality and to enable interpretation of the estimated coefficients as a percentage.

Four MLR models were estimated to identify significant lifestyle and socioeconomic factors associated with global diabetes prevalence. These models were optimized through the minimization of multicollinearity and heteroscedasticity, evaluated using variance inflation factors and a Breusch–Pegan-Godfrey test for heteroscedasticity.

### Literature review

To facilitate the comparison between our results with the existing literature, we confined comparisons to the studies that have used similar risk factors of diabetes in their studies. The literature review sought to promote a comprehensive and interdisciplinary overview of published research including public health studies, economic data, epidemiological analyses, and medical literature.

Peer-reviewed journal articles, reviews, books, databases, government reports, and completed higher-degree research (HDR) theses and dissertations were included in the search criteria while reference materials, magazines, newspaper articles, conference papers, and other grey literature were excluded from the search criteria. Google Scholar, PubMed, and Scopus databases were comprehensively searched using an array of relevant terminology including diabetes; socioeconomic; BMI; tobacco; smoking; alcohol; determinants; correlates; risk; association; epidemiology; and etiology, in multiple permutations, and in combination with Boolean operators. Search parameters were set from between the dates 01/01/2000 up to the date 01/08/2021 to ensure currency. In total, 51 articles including 43 peer-reviewed journal articles, 6 meta-analyses, 1 government report, and 1 database were selected for inclusion.

## 3. Results

**Fig 1:**
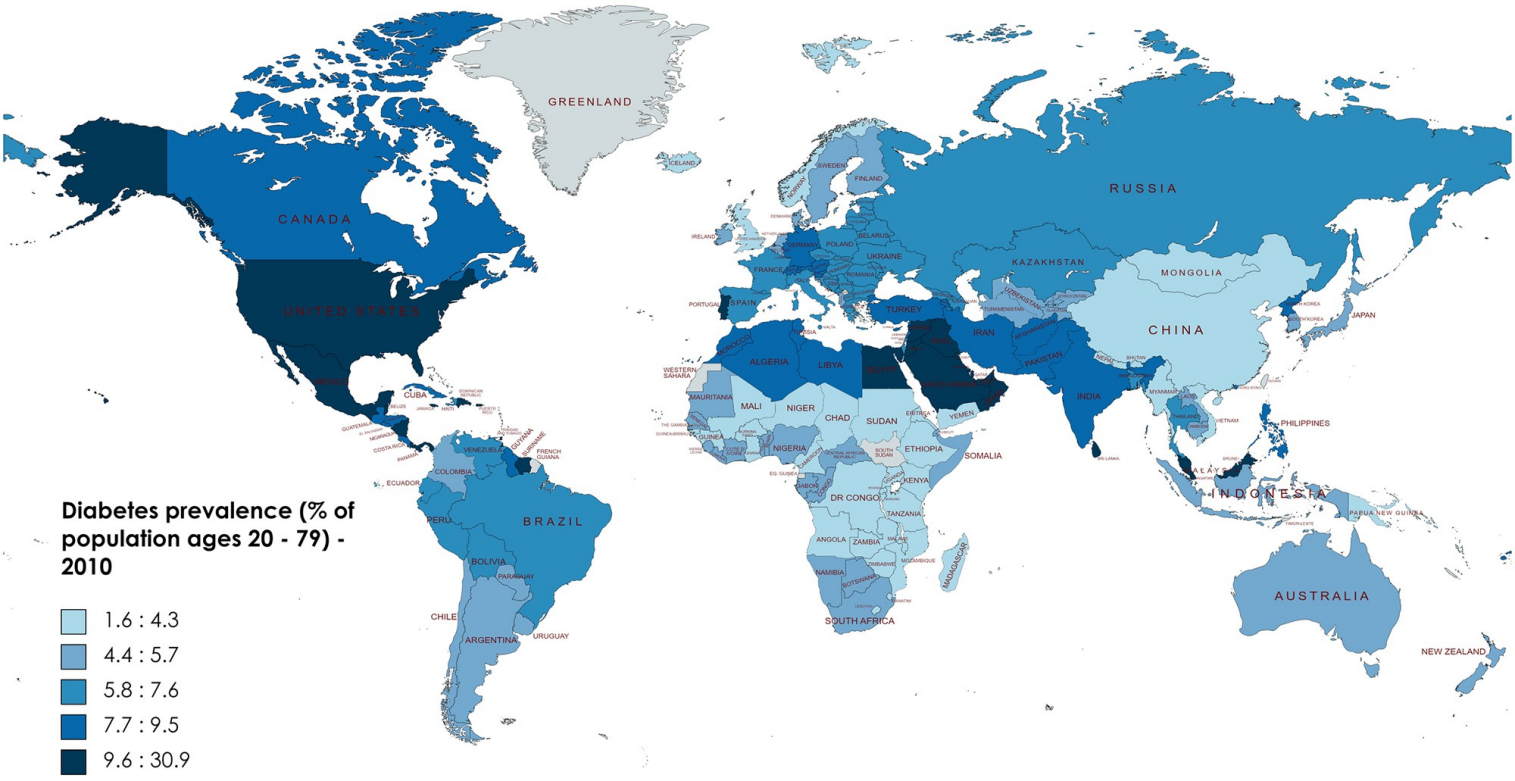
Diabetes prevalence, 2010 (% of population ages 20 to79). The map was created with Mapchart.net.

Intercountry comparisons, showed that diabetes prevalence varied considerably in 2010 ranging from 1.6% in Iceland to 18.7% in the United Arab Emirates [17] (Fig 1).

Summary statistics (mean, variance, and standardized third and fourth moments; skewness and kurtosis statistics) were calculated for the variables included in various models and are given in Table 2. Diabetes prevalence statistics showed a wide range, varying from a minimum value of 0.03% to a maximum of 15.87%. Among the major explanatory variables, the overweight prevalence ranges from 16.5% in India to 75.7% in Tonga, and adult male tobacco prevalence ranges from 8.7% in Ghana to 78% in Timor-Leste. Skewness is a measure of the asymmetry of the distribution of each data series. For a normal distribution, the skewness is zero. According to Table 2 data, the skewness of the diabetes series is 0.18, so we can safely assume a normal distribution in modeling diabetes prevalence in this data panel. In addition, the kurtosis of the diabetes series is less than 3 (the kurtosis of the normal distribution is 3), suggesting that the distribution of the diabetes series is flat relative to the normal distribution. Among the other variables, the population variable seems to suffer from non-normality.

**Table 2:** Summary Statistics of the selected variables across 132 countries.

	DIAB	ALCHO	GNI	LIEX	OWEI	POP	TOBFE	TOBMA	TOBT	UNEM
**Mean**	6.49	6.70	14450	70.29	44.83	47.43	13.31	35.43	24.37	7.64
**Median**	6.68	5.85	4580	73.36	51.90	10.26	10.20	34.00	24.85	6.58
**Max.**	15.87	18.70	88490	82.84	75.70	1337.71	44.10	78.00	52.50	27.31
**Min.**	0.03	1.60	220	45.10	16.50	0.10	0.50	8.70	4.70	0.45
**Std. Dev.**	4.33	3.31	19427	9.31	16.37	14.05	10.70	13.88	10.22	5.64
**Skewness**	0.18	1.15	1.69	-0.69	-0.35	7.09	0.73	0.47	0.28	1.50
**Kurtosis**	1.86	4.49	5.21	2.55	1.67	53.01	2.62	3.11	2.63	5.40

**Fig 2:**
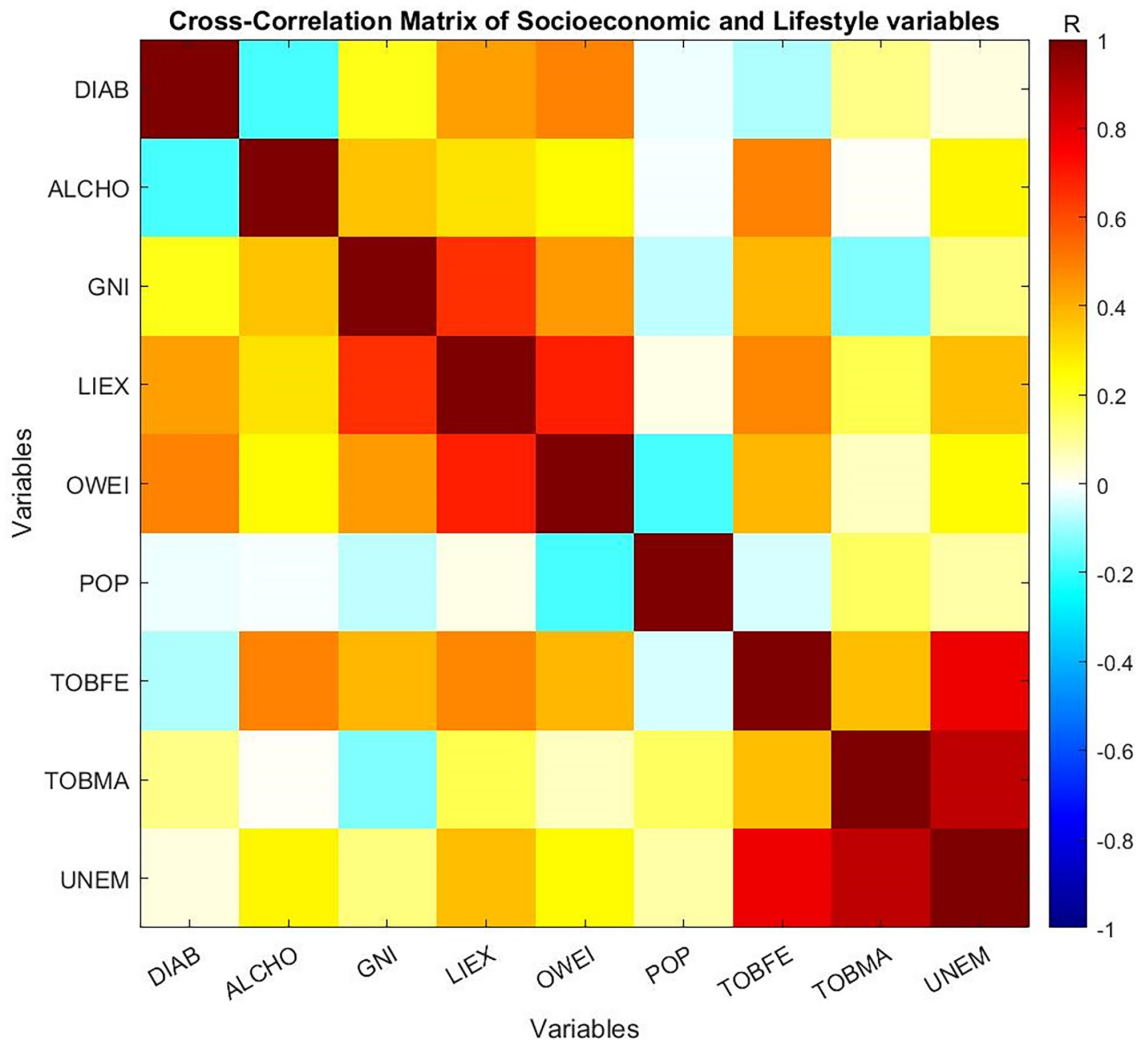
Cross correlation values of socioeconomic and lifestyle variables, for 132 countries and territories, colour coded by the degree of correlation (dark red coloured boxes represents strong positive correlation and dark blue coloured boxes represents strong anti-correlation).

Preliminary analysis of the correlations between variables were assessed using a cross-correlation matrix, (Fig 2 and Table 3). Weak positive correlations were observed between diabetes prevalence and both overweight and life-expectancy variables. Weak negative association were observed between diabetes prevalence and both alcohol consumption and tobacco use in the female population. Strong positive association were observed between unemployment and both the use of tobacco both within the male and female population.

**Table 3:** Cross correlation matrix of socioeconomic and lifestyle variables, for 132 countries and territories.

	DIAB	ALCHO	GNI	LIEX	OWEI	POP	TOBFE	TOBMA	UNEM
DIAB	1.00	-0.18	0.22	0.43	0.49	-0.02	-0.08	0.11	0.03
ALCHO	-0.18	1.00	0.36	0.30	0.25	-0.01	0.49	0.00	0.26
GNI	0.22	0.36	1.00	0.65	0.44	-0.07	0.39	-0.13	0.12
LIEX	0.43	0.30	0.65	1.00	0.68	0.02	0.48	0.17	0.37
OWEI	0.49	0.25	0.44	0.68	1.00	-0.18	0.39	0.06	0.25
POP	-0.02	-0.01	-0.07	0.02	-0.18	1.00	-0.04	0.15	0.08
TOBFE	-0.08	0.49	0.39	0.48	0.39	-0.04	1.00	0.37	0.78
TOBMA	0.11	0.00	-0.13	0.17	0.06	0.15	0.37	1.00	0.87
UNEM	0.03	0.26	0.12	0.37	0.25	0.08	0.78	0.87	1.00

### Model 1: Unrestricted model

The relationship between global diabetes prevalence and the nine explanatory variables are given in Eq (1).


$${\text{DIAB}}_{\text{i}}=\;\propto_{\text{i}}+\beta_{1}{\text{ALCHO}}_{\text{i}}+\beta_{2}{\text{GNI}}_{\text{i}}+\beta_{3}{\text{LIEX}}_{\text{i}}+\beta_{4}{\text{OWEI}}_{\text{i}}+\beta_{5}{\text{POP}}_{\text{i}}+\beta_{6}{\text{TOBFE}}_{\text{i}}+\beta_{7}{\text{TOBMA}}_{\text{i}}+\beta_{8}{\text{TOBT}}_{\text{i}}+\beta_{9}{\text{UNEM}}_{\text{i}}+\varepsilon_{\text{i}}$$ (1)

Adjusted R-squared and F-statistics values are given in Table 4. Three of nine variables were statistically significant (α = 0.05) with diabetes prevalence, which were Log (ALCHO); Log (GNI) and OWEI (Table 4). The coefficient of determination is 0.471, which suggests that the selected variables explain > 47% of the variation in diabetes. However, the majority of slope coefficients of the estimated model is not statistically significant at a 5% level of significance, which raises the possibility of multicollinearity among the explanatory variables [18].

**Table 4:** Model 1 regression results for unrestricted model.

Variable	Coefficient	Std. Error	t-Statistic	Prob.
**C**	-4.7598	2.7588	-1.7253	0.0870
**LOG(ALCHO)**	-0.6346	0.2100	-3.0217	0.0031*
**LOG(GNI)**	0.9089	0.2749	3.3058	0.0012*
**LIEX**	0.0182	0.0465	0.3904	0.6969
**OWEI**	0.0699	0.0202	3.4602	0.0007**
**LOG(POP)**	0.0248	0.1253	0.1978	0.8435
**TOBFE**	3.1228	2.8301	1.1034	0.2720
**TOBMA**	3.2971	2.8270	1.1663	0.2458
**TOBT**	-6.4958	5.6562	-1.1484	0.2530
**UNEM**	-0.0420	0.0422	-0.9955	0.3215
**R^2^ adj**	0.4713		**F-statistic**	13.9739

*P<0.05,

**P<0.01,

***P<0.001

The least-squares estimation method assumes that all nine independent variables are not linearly correlated. If they are correlated, individual coefficients show somewhat inflated joints impacts rather than variable-specific impacts. The level of collinearity between explanatory variables can be measured by looking at the Variance Inflation Factors (VIFs). A high VIF indicates high collinearity. In general, VIF > 5 suggests that the concerned variable is linearly correlated with the other independent variables. Calculated VIFs are shown in Table 5. All tobacco prevalence variables, TOBT, TOBFE, and TOBMA have VIF values that far exceed five (Table 5).

**Table 5:** Variance inflation factors calculated for nine model variables included in the unrestricted model.

Variable	Coefficient Variance	Centered VIF
**LOG(ALCHO)**	4.41E-02	1.33E+00
**LOG(GNI)**	7.56E-02	4.07E+00
**LIEX**	2.20E-03	4.25E+00
**OWEI**	4.00E-04	2.48E+00
**LOG(POP)**	1.57E-02	1.09E+00
**TOBFE**	8.01E+00	2.08E+04
**TOBMA**	7.99E+00	3.49E+04
**TOBT**	3.20E+01	7.57E+04
**UNEM**	1.80E-03	1.28E+00

Collinear variables TOBT and TOBFE were excluded from subsequent estimations because preliminary data analysis showed that female tobacco consumption compared to that of the male is negligible in many countries. LIFEEX was also excluded from further models because the coefficient of LIFEEX has a very small t-value compared to the other eight variables suggesting that LIFEEX is not an important variable to explain the variation in diabetes prevalence in these countries (Table 4). The general-to-specific approach also confirms that the exclusion of LIFEEX has a negligible effect on the statistical properties of the model.

### Model 2: Restricted model

Accordingly, a restricted version of Eq (1) containing six independent variables explaining the diabetes prevalence among the selected countries was developed. The multivariate linear regression estimation is given in Eq 2.


$${\text{DIAB}}_{\text{i}}=\;\propto_{\text{i}}+\beta_{1}{\text{ALCO}}_{\text{i}}+\beta_{2}{\text{GNI}}_{\text{i}}+\beta_{3}{\text{UNEM}}_{\text{i}}+\beta_{4}{\text{OWEIGHT}}_{\text{i}}+\beta_{5}{\text{POP}}_{\text{i}}+\beta_{8}{\text{TOBT}}_{\text{i}}+\varepsilon_{\text{i}}$$ (2)

Eq (2) contains 3 lifestyle and 3 socioeconomic risk factors of diabetes prevalence. The error term ε_i_ is assumed to have constant variance. A Breusch–Pegan-Godfrey test for heteroscedasticity was applied to Model 2. The Breusch–Pegan-Godfrey test convincingly rejects the null hypothesis of homoscedasticity error in the regression. This result is not surprising due to the large differences in socioeconomic factors among the countries in the sample.

As heteroscedasticity is present within the data, the estimated coefficients’ standard errors of coefficients may not be valid. In order to correct for the heteroscedasticity, the original sample are separated into two groups, low volatility and high volatility countries. The primary purpose was to solve the heteroscedasticity problem in the data set. It has been shown that heteroscedasticity leads to incorrect standard errors in the estimated coefficients and incorrect statistical inferences [18]. After a series of trial and error steps, the original data sample of 132 countries were separated into two main groups (low volatility and high volatility countries). The separation was based on the magnitude of the errors from the entire sample. If the absolute value of the prediction error is >2, then those counties are considered high volatility countries. The validity of this grouping was tested through the application of the Breusch–Pegan-Godfrey test for prediction errors coming from these two groups. The Breusch–Pegan-Godfrey test showed no heteroscedasticity within the low and high volatility countries, confirming that the groupings are valid. Thus, the grouping is based on the magnitude of the estimated error. If the absolute value of the error is <2 the country was assigned to the low volatility group, and the rest of the countries are assigned to the high volatility group. 77 countries were assigned to the low volatility group and 55 countries were assigned to the high volatility group. A separate regression equation is estimated for each group; Model 2A uses data from the low volatility group, and Model 2B uses data from the high volatility group. Results are shown in Tables 6 and 7, respectively.

**Table 6:** Model 2A results: Low volatility countries.

Variable	Coefficient	Std. Error	t-Statistic	Prob.
**C**	0.3984	1.5476	0.2574	0.7976
**LOG(ALCHO)**	-0.8524	0.1643	-5.1878	0.0000***
**LOG(GNI)**	0.9177	0.1409	6.5120	0.0000***
**UNEM**	-0.0445	0.0219	-2.0285	0.0463*
**OWEI**	0.0483	0.0128	3.7704	0.0003**
**LOG(POP)**	-0.1892	0.0913	-2.0708	0.0421*
**TOBMA**	0.0262	0.0104	2.5208	0.0140*
**Adjusted R-squared**	0.7537		F-statistic	39.7633

*P<0.05,

**P<0.01,

***P<0.001

**Table 7:** Model 2B results: High volatility countries.

Variable	Coefficient	Std. Error	t-Statistic	Prob.
**C**	3.4355	7.3837	0.4653	0.6438
**LOG(ALCHO)**	-1.0743	0.4000	-2.6858	0.0099**
**LOG(GNI)**	0.1999	0.5015	0.3987	0.6919
**UNEM**	-0.1993	0.1269	-1.5713	0.1227
**OWEI**	0.0733	0.0426	1.7192	0.0920
**LOG(POP)**	0.1668	0.2734	0.6102	0.5446
**TOBMA**	-0.0293	0.0418	-0.7015	0.4864
**Adjusted R-squared**	0.1752		F-statistic	2.9119

*P<0.05,

**P<0.01,

***P<0.001

Estimation results suggest that the specification used to explain diabetes prevalence is more suited to low volatility countries but less suitable to the high volatility countries. To illustrate, based on the adjusted R^2^ value, about 75% of the variation in diabetes prevalence can be explained for the low volatility group by using the six explanatory variables included in the estimation. A high F-statistic value given in Table 6 further confirms the goodness of fit in modelling diabetes prevalence in low volatility countries. On the other hand, the model specification performs rather poorly for the high volatility group. Only about 17% of the variation in diabetes prevalence can be explained with a small F-statistic value indicating that the model is a considerably poor fit compared to that of the low volatility group. The statistical insignificance of the majority of the independent variables further confirms the weakness of the model specification. To illustrate, according to Model 2B results, five out of six independent variables turn out to be statistically insignificant at the 5% level of significance. On the other hand, all six variables are statistically significant in low volatility countries. As a result, we focus on Model 2A results that we estimated from 77 low volatility countries and leave out Model 2B results based on 55 high volatility countries in the subsequent analysis.

#### Spatial distribution of low volatility countries

**Fig 3:**
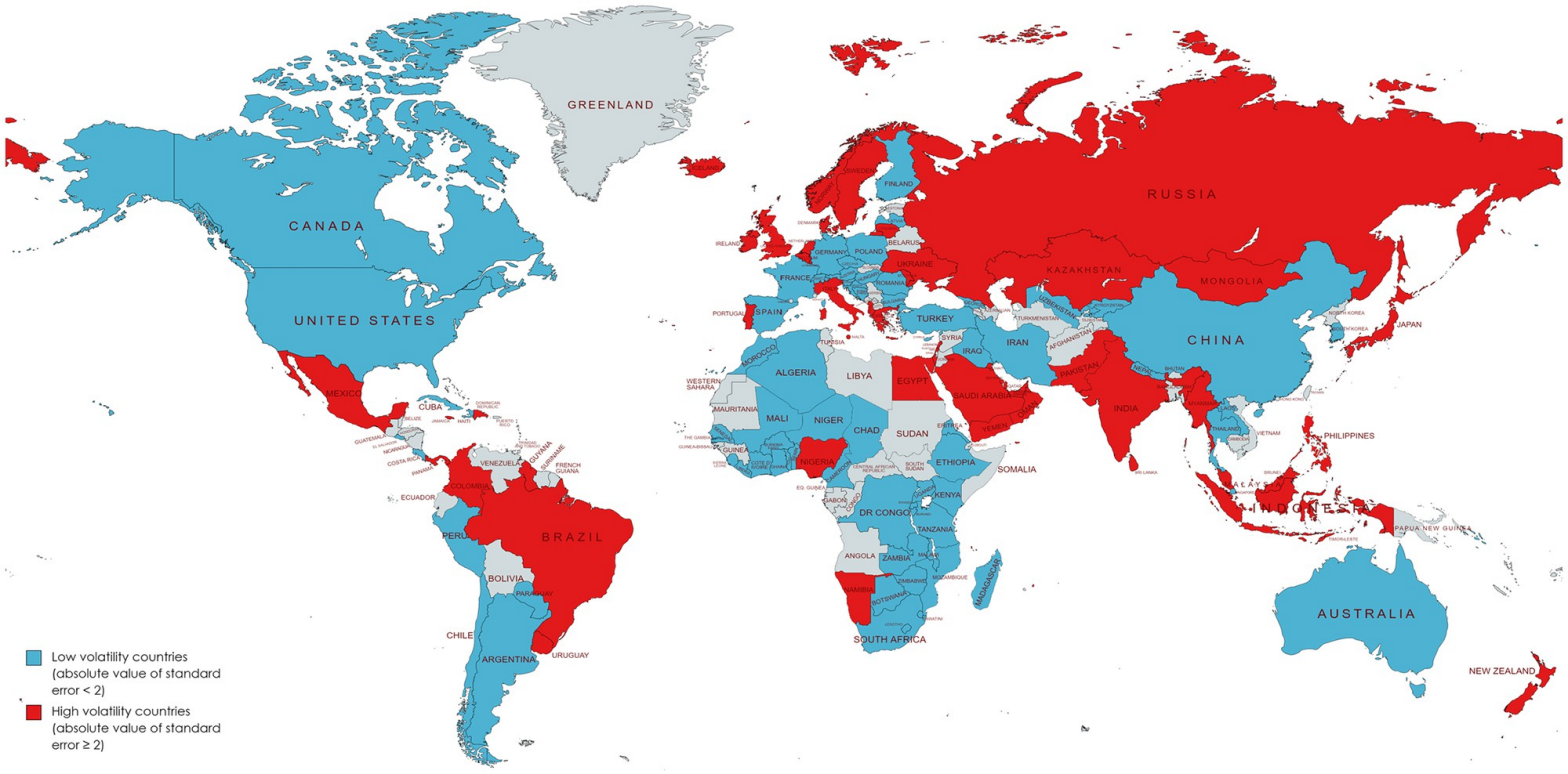
Spatial distribution of low volatility countries, based on partitioning of absolute value of standard errors (if SE<2, low volatility country, if SE≥2, high volatility country). The map was created with Mapchart.net.

The spatial distribution of the low and high volatility countries are shown in blue and red respectively on the map below (Fig 3).

A number of high and upper middle income countries were excluded from the analysis due to high heteroscedasticity including countries within Northern Europe; South West Asia; Northern Asia and South America. Most of the lower income and lower middle income countries were included apart from a few countries within Southern Asia and central Europe.

## 4. Discussion

### Diabetes

Diabetes mellitus is broadly categorised into three types according to underlying etiology and clinical presentation: type 1 diabetes mellitus (T1DM), type 2 diabetes mellitus (T2DM), and gestational diabetes mellitus (GDM) [19–21]. The pathophysiology of T1DM is predominantly considered to be autoimmune destruction of pancreatic β-cells. This usually develops during childhood and is considered rare among adults [19]. On the other hand, the pathophysiology of T2DM is more complicated and involves impaired insulin secretion from pancreatic β-cells as well as peripheral insulin resistance [20]. While T2DM is underpinned by a range of non-modifiable risk factors such as age, ethnic background, and genetic predisposition, there are several well-established modifiable driving forces such as low socioeconomic status (SES) excess body mass, inadequate physical activity, tobacco smoking, and alcohol intake that have been found to attenuate the risk of T2DM [6, 20]. The underlying pathophysiology of GDM is similar to T2DM in terms of pancreatic β-cell inadequacy in conjunction with insulin resistance, however in this situation, the insulin resistance is exacerbated by placental hormones which block the action of insulin [22]. GDM is clinically defined as hyperglycaemia that first develops or is diagnosed in late pregnancy [22]. In general, GDM disappears after the pregnancy as placental hormones return to baseline levels [22]. However, research has indicated GDM to be a strong predictor of future T2DM onset for both the mother and the child [22].

Modifiable risk factors involved in the pathogenesis of T2DM are attractive targets for public health interventions. Unfortunately, the nature of cross-sectional research and survey data that has been used to investigate the associations of various risk factors with incident diabetes have focused on the prevalence of diabetes regardless of the subtype [23, 24]. While this may indeed be a limitation to the conclusions that are drawn from this research, prevalence data from a recent US population-based study reported type 1 and 2 diabetes to consist of 5.6% and 91.2% of the total diabetes cases respectively [21]. Other studies investigating the global prevalence of diabetes subtypes have reported similar findings [1, 2]. Given the overwhelming dominance of T2DM in the total cases of DM, it is unlikely that the inability to distinguish between disease subtypes would impact the results to any significant degree. For the purposes of this discussion, the terms diabetes, type 2 diabetes mellitus, and T2DM are used interchangeably unless specified otherwise.

### Socioeconomic status and diabetes

Our results show that as far as socioeconomic factors are concerned, the per capita income variable is statistically significant and positively related to diabetes prevalence. Among all explanatory variables included in model 2A, the per capita income seems to exert the highest impact on diabetes prevalence. For example, a 1-percent increase in per capita income would lead to a 0.92 percent increase in diabetes prevalence. The magnitude and the direction of the income on diabetes prevalence are not surprising. The existing studies suggest that high-income countries tend to have higher levels of diabetes prevalence compared to poorer nations [2].

The other two socioeconomic factors (total population and unemployment) are also statistically significant but negatively correlate with diabetes prevalence. Patterning of socioeconomic risk factors has been widely undertaken and reported in the literature. These studies have historically used a diverse range of proxy measures to account for socioeconomic status (SES) including education level, income, and occupation which have made comparisons between these studies difficult. In a community sample of 6147 diabetes-free adults in Alameda county, each of these variables was taken as a proxy measure for SES and analyzed for an association with downstream type 2 diabetes over a 34-year study period [14]. Although all three proxy measures showed a positive association, low educational attainment was found to be the strongest predictor of incident type 2 diabetes [14]. Interestingly, these associations were attenuated by covariate adjustment for confounding variables; obesity and overweight BMI being the strongest mediators of this association [14]. A similar study with two of the same measures of SES (education level and occupation) along with poverty income ratio (PIR) as a third variable, followed a cohort of 10849 adults who were free of diabetes for a mean follow-up time of 10 years [25]. Among both men and women, there was an inverse association of diabetes with education level and PIR, however, occupational status was only inversely associated with diabetes prevalence in women [25]. Similar gender differences were also found in a Canadian community study with females from a lower SES background (as measured by income level), having a higher risk of developing T2D compared to men [26]. Reasons for these gender differences remain unclear although some evidence has found women of higher SES to be significantly more active than those of lower SES [26]. This pattern was not seen in men which could explain some of the variation [26]. These results are consistent with findings from a nationally representative Korean population study which found lower educational attainment to be an independent risk factor for T2D [27]. Moreover, individuals with the lowest income level were more likely to have type 2 diabetes than those with the highest income level [27]. The importance of education levels as a risk factor for T2D is possibly explained by the fact that lower educational attainment limits knowledge uptake across the life course and links unhealthy behaviours with environmental exposures, accumulating downstream risk [27].

A major confounding variable when determining the association between SES and the diabetes prevalence of a population is the effect that regional deprivation may have [11]. Regional deprivation is often used as a proxy for individual SES [11]. A German pooled analysis of five population-based studies has demonstrated that the deprivation status of a place of residence is independently associated with individual socio-economic factors such as education level [11]. However, since regional deprivation is linked to area-level indicators such as employment, there is some overlap between measures of deprivation, and unemployment and income level variables which are proxy measures of SES [11]. Consistent with previous research, survey data from the Basque Country in Spain have shown the prevalence of type 2 diabetes to be independently higher in patients of lower SES and in patients who have experienced a higher level of regional deprivation [12].

Together these studies suggest that the inverse association between SES and diabetes that has been widely reported in the literature is not merely a downstream function of diabetes, but rather, reflects a distinct increase in the risk of developing diabetes in populations with a low SES that is independent of the regional deprivation factor of a population [28]. Although SES is not traditionally considered a modifiable risk factor, there is a substantial scope for public health interventions to address underlying determinants of low SES such as barriers in access to education, employment, and physical activity [29].

Our results show that the negative relationship between unemployment and diabetes prevalence is not inconsistent with the literature. Casual observation suggests that countries with higher populations have a lower diabetes prevalence, but this needs further investigation. Our results indicate that an increase in the unemployment rate decreases diabetes prevalence. This may be due to an increasingly serviced or sedentary lifestyle (including watching TV; sitting at work and other sitting; increased mechanization and driving) in the employed population [30]. Secondly a reduction in unemployment (or an increase in average income) would result in higher levels of spending on discretionary foods (high caloric with poor nutritional value), which may result in an increase in the prevalence of diabetes. Conversely an increase in unemployment (or reduction in net income) may reduce the proportion of income spent on these discretionary foods [31]. As far as the socioeconomic factors are concerned, there is no unanimous agreement on how they affect diabetes prevalence. However, the socioeconomic factors might contribute to the development of type 2 diabetes through processes involving lack of access to health care services, healthy foods, places to exercise, and occupational opportunities, leading to unhealthy lifestyle practices, so that the impacts mostly could be indirect [32].

### Body mass index and diabetes

Another commonly cited correlate of diabetes is body mass index (BMI) which is defined as the ratio of weight (kg) divided by the square of height (m^2^). Several studies which have assessed the relationship between socioeconomic factors and diabetes have consistently found BMI to independently explain between 32% and 45% of this association [29, 33, 34]. This is expected with the underlying pathophysiology of type 2 diabetes which has been linked to overnutrition, but it may also be explained by lower levels of access to facilitators of physical activity such as gyms, community parks, and recreational facilities [35].

Our results show that as anticipated overweight is a significant risk factor for diabetes. The coefficient of overweight is significant at virtually any level of significance and positively impacts diabetes prevalence. Specifically, results suggest that a 1-percent increase in overweight prevalence would lead to an average 0.05 percent increase in diabetes prevalence assuming other factors remain fixed.

Furthermore, evidence has overwhelmingly shown increased diabetes incidence to be strongly associated with higher body mass index (BMI) levels [8–10, 29, 33, 36, 37]. However, the relationship between body mass and diabetes has been largely attributed to the proportion and distribution of visceral (within the abdominal cavity) body fat [8, 10, 36–38]. There are several limitations to using BMI as a health indicator. For instance, it does not account for individual differences in body composition, racial and gender differences, or distinguish between subcutaneous and more harmful visceral distributions of body fat. Nonetheless, BMI maintains clinical utility as it is the most economical and practical approach to identify individuals who may be at an increased risk of metabolic complications [38, 39].

Existing research indicates that the association between BMI and diabetes has been found to diminish substantially as BMI decreases towards a normal range (18.5–24.9 kg/m^2^). For this reason, some previous studies have defined a novel measure for overweight which includes all BMI levels greater or equal to the upper (25 kg/m^2^) bounds of the healthy BMI level for the purpose of analysis [34]. However, this figure has been the subject of some debate since lower BMI levels have been found to have better sensitivity and specificity for metabolic risk factors in certain populations [38]. More recent evidence has suggested that the upper limit of normal for the BMI of South Asian, Black, Chinese, and Arab populations should be reduced to account for the higher relative proportion of visceral fat in these ethnic groups [7]. Current policy recommendations encourage setting a threshold BMI of ≥ 23 kg/m^2^ to denote “overweight” in Chinese Asians [38]. Similarly, clinical diagnostic recommendations in India consider a BMI of ≥ 23 kg/m^2^ as overweight [39]. It is commonly known that BMI alone is a poor indicator of metabolic and cardiovascular risk stratification. It is recommended that the ethnicity-specific BMI classification is considered in combination with anthropometric measures such as waist circumference and waist-to-hip ratio to increase predictive sensitivity and specificity for the downstream onset of diabetes [7, 39].

The Whitehall II cohort study analysed a set of modifiable risk factors in London-based civil servants including BMI, smoking, and alcohol in terms of their contribution to social inequalities seen in the incidence of type 2 diabetes [33]. BMI was found to be the most important factor contributing to the onset of diabetes and independently explained up to 23% of the inequalities contributing to type 2 diabetes [33]. When hyperlipidaemia and health behaviours were considered along with BMI, up to 53% of the relationship was explained [33]. Similarly, a hospital-based study from urban Ghana found diabetes to primarily affect low SES, high BMI patients with central adiposity and accompanying hyperlipidaemia [9]. This is in contrast to the findings of several Indian studies that report obesity (defined in India as BMI ≥ 25 kg/m^2^) and associated metabolic risk factors as more common in higher SES groups. This is noteworthy as it may indicate the early stages of epidemiological transition [40–42].

A three-tier staging system has been used to describe the epidemiological transition of populations towards the stratification of obesity that exists in developed countries [42]. Stage 1 of the epidemiological transition is defined by a higher prevalence of obesity in women than men, and a greater prevalence in high SES than low SES populations. Stage 2 of transition sees the narrowing of the disparities between genders and SES groups. The third and final stage of the epidemiological transition occurs when lower SES groups overtake higher SES groups in terms of the prevalence of obesity. Identification of countries in the early stages of the epidemiological transition may allow policymakers to predict at-risk populations and intervene with proactive solutions to attenuate the transition.

Racial differences within populations have also been identified in the association of BMI with diabetes incidence [8]. An American cohort study investigated adults aged 40 to 79 from 12 southern American states to investigate the rates of incident diabetes in a racially diverse population with a high prevalence of obesity. As expected, there was strong evidence to suggest that elevated BMI was associated with higher frequencies of diabetes incidence in both black and white racial groups. However, the incidence of diabetes was found to be twice as high among the black population with normal BMI than in the corresponding white population [8]. Curiously, this difference was attenuated as the BMI increased into overweight and obese territories with the estimated five-year probability of developing diabetes estimated at 20% for both groups when predicted at the morbidly obese level (BMI ≥ 40 kg/m^2^) [8]. Disparities in the onset of diabetes have been proposed to arise primarily from differences in the environments that African Americans and white populations may reside in; when these groups live in similar risk environments, disparities in diabetes and wider health outcomes are ameliorated [43]. These findings encourage reductions in structural segregation and promote policy implementation that would prioritise the allocation of resources to lower SES areas.

### Smoking and diabetes

There is an increasing pool of evidence suggesting a strong relationship between tobacco smoking and incident diabetes [13, 44–46]. Our results show that tobacco consumption exerts a positive and statistically significant impact on diabetes prevalence. For example, a 1-percent increase in tobacco prevalence would increase diabetes prevalence by about 0.2 percent. Evidence from the literature shows that the increased risk of incident diabetes exists not only for active smoking but also for those who are exposed to smoking passively [46]. A community-based cross-sectional study in Saudi Arabian adults found that this relationship exists only with smoked and not smokeless tobacco products [47]. This is important to consider since much of the available research does not specify the nature of the consumption of tobacco and this may explain some conflicting findings.

Landmark data from the Insulin Resistance Atherosclerosis prospective cohort study determined the relationship between smoking categories (never, former, and current) and the incident 5-year type-2 diabetes onset in American adults who were free of diabetes at baseline [45]. After adjusting for external variables, never smokers were 2.6 times less likely to develop incident diabetes than current smokers and 1.3 times less likely to develop incident diabetes than former smokers [45]. Encouragingly, a similar study analysed data from the Women’s Health Initiative observational and prospective cohort studies, found the risk of developing incident diabetes decreased as the time since quitting increased and was no different to that of never-smokers after a period of cessation of 10 years [44, 45]. Both studies found an independent, inverse association between smoking and BMI which may attenuate some of the metabolic risk [44, 45]. Due to the strong relationship between body mass and diabetes, it is likely that residual confounding variation exists and so the true association of smoking with incident diabetes may be even higher.

Curiously, several studies have reported a protective effect of smoking on incident diabetes [15, 16]. A follow-up study of Japanese men aged 30–59 at baseline found a reduction in the risk of incident diabetes in lean men [16]. Similarly, a prospective cohort study of Turkish adults found women showed a lower risk of developing diabetes albeit without improvements in mortality or overall health benefit [15]. This has been primarily explained by the associated reduction in appetite and rise in metabolic rate seen among smokers [15, 16]. In conjunction with this research, there is an increasing body of literature that suggests smoking cessation can be accompanied by substantial weight gain which may increase the risk of diabetes [48, 49]. Significant weight gain of approximately 5kg, 10 years after quitting smoking, has been reported in the literature compared to counterparts who continued to smoke [48]. Concerningly, it is overweight smokers that tend to gain the most weight on cessation of smoking. Overweight smokers that continue to smoke are likely to remain stable or lose weight. If weight gain following smoking cessation were to lead to an increased risk of diabetes onset, this would represent a significant challenge for upstream intervention since addressing one metabolic risk factor may lead to an exacerbation of another. However, a nationally representative cohort study of Australian adults examined this issue and found that despite the weight gain associated with smoking cessation, people who quit smoking had a significantly lower risk of death than those who continued to smoke [49]. Furthermore, neither the weight change nor the resultant change in BMI was associated with an increase in incident diabetes. These findings raise the attractiveness of smoking cessation interventions as a target to curb the downstream incidence of diabetes [49].

### Alcohol intake and diabetes

The low volatility model from our analysis suggests that alcohol intake exerts a significantly negative impact on diabetes prevalence (Model 2A, Table 6). Specifically, a 1-percent increase in alcohol consumption decreases the diabetes prevalence by 0.85 percent. This is a counterintuitive result, but this is not the first time a negative relationship is identified between alcohol consumption and diabetes prevalence variables. Current evidence for the relationship between alcohol consumption and incident diabetes is somewhat ambiguous and remains controversial due to inconsistent results across studies [50]. A large proportion of the literature suggests that a low to moderate alcohol intake is inversely related to diabetes onset [50–54]. Several studies have found this relationship to be more pronounced in female populations [52, 55]. For example, a multicentre prospective case-cohort performed with data across eight European countries found that among participants who consumed moderate levels of alcohol, only women experienced a lower risk of type 2 diabetes [55]. However, a large meta-analysis of 20 cohort studies concluded that moderate alcohol intake is protective in both men and women with the optimal protective intake at 22g/day of alcohol in men and 24g/day alcohol for women [50]. High levels of alcohol intake beyond 50g/day for women and 60g/day in men achieved significance as a positive risk factor for incident diabetes and remained deleterious beyond this point [50]. A 20-year follow-up of the Finnish Twin Cohort study reported similar findings with moderate consumption of alcohol (5–29.9 g/day in men and 5–19.9 g/day in women) associated with a reduced incidence of diabetes when compared to those with low consumption (< 5g/ day) of alcohol [52].

The inverse relationship between alcohol consumption and diabetes was also found to be more pronounced in overweight than in normal-weight populations [50, 54, 55]. The protective effect of alcohol consumption which is more visible in women and overweight subjects is potentially explained by studies that have shown alcohol to be associated with enhanced insulin sensitivity [56]. Women have a genetically greater proportion of fat mass compared to men. It is plausible that the increased insulin sensitivity conferred by alcohol consumption could offset adiposity-induced insulin resistance in both women and overweight populations [56]. This explanation is supported by the findings of the Finnish Twin Cohort study which reported that moderate alcohol consumption was associated with a 30–40% reduction in the risk of T2D in overweight (BMI ≥ 25kg/m^2^) men and women and no corresponding reduction in risk in lean or normal weight (BMI ≤ 25kg/m^2^) men who would not have high levels of adiposity-induced insulin resistance [52, 56].

In fact, existing studies suggest a U-shape impact of alcohol consumption on diabetes prevalence [50, 51, 55]. At moderate levels, alcohol consumption exerts a negative impact on diabetes prevalence, but higher levels of alcohol consumption would lead to a higher level of diabetes prevalence. A U-shaped relationship between alcohol consumption and type 2 diabetes was confirmed by a meta-analysis of 20 cohort studies [32]. However, caution was given to the interpretation of the U-shape association because the link is not as simple as it looks [32]. As the article states, “alcohol consumption was more strongly associated with reduced risk for type 2 diabetes among overweight compared with normal-weight men and women.” It is possible that the association might be coming from the link between body fatness and alcohol relationship rather than directly from alcohol consumption. Although a lesser number of studies have also suggested a J–shaped association (beneficial when consumed sparingly) relationship between these variables [53]. In any case, this is important since traditional public health messaging oriented around reduction, restriction, and limitation may be difficult to promote and enforce in U and J-shaped associations, particularly when compared with determinants that have linear relationships with health outcomes.

## 5. Strengths and limitations

To our knowledge, this is the first study that investigated relationships between the prevalence of diabetes and lifestyle and socioeconomic risk factors globally. Although numerous studies have attempted to uncover socioeconomic and lifestyle-based risk factors affecting the prevalence of diabetes, the majority of these have used survey-based, micro-level data from hospitals, cities, or regions of a country [7–16]. As these previously published studies were not approached from a global perspective, statistical inferences cannot be reliably generalized to the rest of the world. Our study may inspire reflections from policy makers within government, health services and economic industries at a global level.

There are some limitations associated with our study. First, the nature of cross-sectional research and survey data that has been used to investigate the associations of various risk factors with incident diabetes have focused on the prevalence of diabetes regardless of the subtype [23, 24]. While this may indeed be a limitation to the conclusions that are drawn from this research. prevalence data from a recent US population-based study reported type 1 and 2 diabetes to consist of 5.6% and 91.2% of the total diabetes cases respectively [21]. Other studies investigating the global prevalence of diabetes subtypes have reported similar findings [1, 2]. Given the overwhelming dominance of T2DM in the total cases of DM, it is unlikely that the inability to distinguish between disease subtypes would impact the results to any significant degree. Secondly, the significant reduction in explanatory variables and countries / territories due to data availability and high heteroscedasticity respectively means that statistically significant determinants of global diabetes prevalence could have been omitted, due to the amount of missing data. Thirdly, our study looks at associations but does not investigate causality in the relationship between health and socioeconomic indicators and diabetes prevalence. Fourth, country level data may conceal discrepancies between subnational entities in terms of outcomes and predictors.

## 6. Conclusion

Statistically significant global socioeconomic determinants of diabetes include per capita income, total population and unemployment rate. Statistically significant global lifestyle determinants of diabetes include tobacco consumption; overweight prevalence and alcohol consumption. Per capita income; tobacco consumption and overweight determinants increased with diabetes prevalence, whereas unemployment; total population and alcohol consumption decreased with diabetes prevalence. These observations suggest that there are modifiable risk factors which are consistent at both the micro and macro-level (tobacco consumption and overweight), for which global targeted interventions can be considered. There are determinant such as total population and unemployment which cannot be easily modified and required further investigation to reveal underlying factors associated with their outcomes. Finally, there are risk factors such as alcohol consumption which have a non-linear association with diabetes at the micro-level. This non-linear relationships warrants further research to determine global cut-off points at which alcohol becomes less protective against diabetes. Although this research is limited by missing data and heteroscedasticity, the use of cross-sectional based study for country level aggregate data is a critical tool that should be considered when making global joint strategies or policies against diabetes in both data analysis and decision making.

## References

[1] Lin X, Xu Y, Pan X, Xu J, Ding Y, Sun X, et al. Global, regional, and national burden and trend of diabetes in 195 countries and territories: an analysis from 1990 to 2025. Sci Rep. 2020;10. doi: 10.1038/s41598-020-71908-9 32901098 PMC7478957

[2] Saeedi P, Petersohn I, Salpea P, Malanda B, Karuranga S, Unwin N, et al. Global and regional diabetes prevalence estimates for 2019 and projections for 2030 and 2045: Results from the International Diabetes Federation Diabetes Atlas, 9th edition. Diabetes Res Clin Pract. 2019;157. doi: 10.1016/j.diabres.2019.107843 31518657

[3] Taher N, Huda MSB, Chowdhury TA. COVID-19 and diabetes: What have we learned so far? Clin Med J R Coll Physicians London. 2020;20: E87–E90. doi: 10.7861/clinmed.2020-0261 32628128 PMC7385778

[4] Holman N, Knighton P, Kar P, O’Keefe J, Curley M, Weaver A, et al. Risk factors for COVID-19-related mortality in people with type 1 and type 2 diabetes in England: a population-based cohort study. Lancet Diabetes Endocrinol. 2020;8: 823–833. doi: 10.1016/S2213-8587(20)30271-0 32798471 PMC7426091

[5] Bassett MT. Diabetes is epidemic. Am J Public Health. 2005;95: 1496. doi: 10.2105/ajph.95.9.1496 16211717 PMC1449387

[6] Timpel P, Harst L, Reifegerste D, Weihrauch-Blüher S, Schwarz PEH. What should governments be doing to prevent diabetes throughout the life course? Diabetologia. 2019;62: 1842–1853. doi: 10.1007/s00125-019-4941-y 31451873

[7] Caleyachetty R, Barber TM, Mohammed NI, Cappuccio FP, Hardy R, Mathur R, et al. Ethnicity-specific BMI cutoffs for obesity based on type 2 diabetes risk in England: a population-based cohort study. Lancet Diabetes Endocrinol. 2021;9: 419–426. doi: 10.1016/S2213-8587(21)00088-7 33989535 PMC8208895

[8] Conway BN, Han X, Munro HM, Gross AL, Shu XO, Hargreaves MK, et al. The obesity epidemic and rising diabetes incidence in a low-income racially diverse southern US cohort. PLoS One. 2018;13. doi: 10.1371/journal.pone.0190993 29324894 PMC5764338

[9] Danquah I, Bedu-Addo G, Terpe KJ, Micah F, Amoako YA, Awuku YA, et al. Diabetes mellitus type 2 in urban Ghana: Characteristics and associated factors. BMC Public Health. 2012;12. 22429713 10.1186/1471-2458-12-210 PMC3364878

[10] Gupta S, Bansal S. Does a rise in BMI cause an increased risk of diabetes?: Evidence from India. PLoS One. 2020;15: e0229716. doi: 10.1371/journal.pone.0229716 32236106 PMC7112218

[11] Jacobs E, Tönnies T, Rathmann W, Brinks R, Hoyer A. Association between regional deprivation and type 2 diabetes incidence in Germany. BMJ Open Diabetes Res Care. 2019;7. doi: 10.1136/bmjdrc-2019-000857 31908802 PMC6936410

[12] Larrañaga I, Arteagoitia JM, Rodriguez JL, Gonzalez F, Esnaola S, Piniés JA. Socio-economic inequalities in the prevalence of Type 2 diabetes, cardiovascular risk factors and chronic diabetic complications in the Basque Country, Spain. Diabet Med. 2005;22: 1047–1053. doi: 10.1111/j.1464-5491.2005.01598.x 16026371

[13] Liu X, Bragg F, Yang L, Kartsonaki C, Guo Y, Du H, et al. Smoking and smoking cessation in relation to risk of diabetes in Chinese men and women: a 9-year prospective study of 0·5 million people. Lancet Public Heal. 2018;3: e167–e176. doi: 10.1016/S2468-2667(18)30026-4 29548855 PMC5887081

[14] Maty SC, Everson-Rose SA, Haan MN, Raghunathan TE, Kaplan GA. Education, income, occupation, and the 34-year incidence (1965–99) of Type 2 diabetes in the Alameda County Study. Int J Epidemiol. 2005;34: 1274–1281. doi: 10.1093/ije/dyi167 16120636 PMC3172611

[15] Onat A, Özhan H, Esen AM, Albayrak S, Karabulut A, Can G, et al. Prospective epidemiologic evidence of a “protective” effect of smoking on metabolic syndrome and diabetes among Turkish women-Without associated overall health benefit. Atherosclerosis. 2007;193: 380–388. doi: 10.1016/j.atherosclerosis.2006.07.002 16926017

[16] Nagaya T, Yoshida H, Takahashi H, Kawai M. Heavy Smoking Raises Risk for Type 2 Diabetes Mellitus in Obese Men; But, Light Smoking Reduces the Risk in Lean Men: A Follow-up Study in Japan. Ann Epidemiol. 2008;18: 113–118. doi: 10.1016/j.annepidem.2007.07.107 18083537

[17] The World Bank. Health nutrition and population statistics. data, editor. In: World Bank Washington, DC [Internet]. Washington, {DC}: World Bank.; 2021. https://datatopics.worldbank.org/health/home

[18] Brooks C. Introductory Econometrics for Finance. 4th ed. Cambridge: Cambridge University Press; 2019. doi: 10.1017/9781108524872

[19] Katsarou A, Gudbjörnsdottir S, Rawshani A, Dabelea D, Bonifacio E, Anderson BJ, et al. Type 1 diabetes mellitus. Nat Rev Dis Prim. 2017;3: 1–17. doi: 10.1038/nrdp.2017.16 28358037

[20] Chatterjee S, Khunti K, Davies MJ. Type 2 diabetes. Lancet. 2017;389: 2239–2251. doi: 10.1016/S0140-6736(17)30058-2 28190580

[21] Xu G, Liu B, Sun Y, Du Y, Snetselaar LG, Hu FB, et al. Prevalence of diagnosed type 1 and type 2 diabetes among US adults in 2016 and 2017: Population based study. BMJ. 2018;362. doi: 10.1136/bmj.k1497 30181166 PMC6122253

[22] Noctor E. Type 2 diabetes after gestational diabetes: The influence of changing diagnostic criteria. World J Diabetes. 2015;6: 234. doi: 10.4239/wjd.v6.i2.234 25789105 PMC4360417

[23] Geiss LS, Wang J, Cheng YJ, Thompson TJ, Barker L, Li Y, et al. Prevalence and incidence trends for diagnosed diabetes among adults aged 20 to 79 years, United States, 1980–2012. JAMA—J Am Med Assoc. 2014;312: 1218–1226. doi: 10.1001/jama.2014.11494 25247518

[24] Wang L, Gao P, Zhang M, Huang Z, Zhang D, Deng Q, et al. Prevalence and ethnic pattern of diabetes and prediabetes in China in 2013. JAMA—J Am Med Assoc. 2017;317: 2515–2523. doi: 10.1001/jama.2017.7596 28655017 PMC5815077

[25] Robbins JM, Vaccarino V, Zhang H, Kasl S V. Socioeconomic status and diagnosed diabetes incidence. Diabetes Res Clin Pract. 2005;68: 230–236. doi: 10.1016/j.diabres.2004.09.007 15936465

[26] Tang M, Chen Y, Krewski D. Gender-related differences in the association between socioeconomic status and self-reported diabetes. Int J Epidemiol. 2003;32: 381–385. doi: 10.1093/ije/dyg075 12777423

[27] Hwang J, Shon C. Relationship between socioeconomic status and type 2 diabetes: Results from Korea National Health and Nutrition Examination Survey (KNHANES) 2010–2012. BMJ Open. 2014;4. doi: 10.1136/bmjopen-2014-005710 25138810 PMC4139629

[28] Agardh E, Allebeck P, Hallqvist J, Moradi T, Sidorchuk A. Type 2 diabetes incidence and socio-economic position: a systematic review and meta-analysis. Int J Epidemiol. 2011;40: 804–818. doi: 10.1093/ije/dyr029 21335614

[29] Lee TC, Glynn RJ, Peña JM, Paynter NP, Conen D, Ridker PM, et al. Socioeconomic status and incident type 2 diabetes mellitus: Data from the women’s health study. PLoS One. 2011;6. doi: 10.1371/journal.pone.0027670 22194788 PMC3237410

[30] Hu FB. Globalization of Diabetes. Diabetes Care. 2011;34: 1249–1257. doi: 10.2337/dc11-0442 21617109 PMC3114340

[31] Penrose G, Cava G La. Job Loss, Subjective Expectations and Household Spending. 2021 [cited 27 Dec 2021].

[32] Li Y, Ley SH, Tobias DK, Chiuve SE, VanderWeele TJ, Rich-Edwards JW, et al. Birth weight and later life adherence to unhealthy lifestyles in predicting type 2 diabetes: Prospective cohort study. BMJ. 2015;351. doi: 10.1136/bmj.h3672 26199273 PMC4510778

[33] Stringhini S, Tabak AG, Akbaraly TN, Sabia S, Shipley MJ, Marmot MG, et al. Contribution of modifiable risk factors to social inequalities in type 2 diabetes: Prospective Whitehall II cohort study. BMJ. 2012;345. doi: 10.1136/bmj.e5452 22915665 PMC3424226

[34] Balasooriya NN, Bandara JS, Rohde N. The intergenerational effects of socioeconomic inequality on unhealthy bodyweight. Heal Econ (United Kingdom). 2021;30: 729–747. doi: 10.1002/hec.4216 33438790

[35] Gupta D, B. Krueger C, Lastra G. Over-nutrition, Obesity and Insulin Resistance in the Development of b-Cell Dysfunction. Curr Diabetes Rev. 2012;8: 76–83. doi: 10.2174/157339912799424564 22229253

[36] Sonmez A, Yumuk V, Haymana C, Demirci I, Barcin C, Kıyıcı S, et al. Impact of obesity on the metabolic control of type 2 diabetes: Results of the Turkish nationwide survey of glycemic and other metabolic parameters of patients with diabetes mellitus (TEMD Obesity study). Obes Facts. 2019;12: 167–178. doi: 10.1159/000496624 30893706 PMC6547285

[37] Akbarzadeh A, Salehi A, Molavi Vardanjani H, Poustchi H, Gandomkar A, Fattahi MR, et al. Epidemiology of Adult Diabetes Mellitus and its Correlates in Pars Cohort Study in Southern Iran. Arch Iran Med. 2019;22: 633–639. 31823628

[38] Hsu WC, Araneta MRG, Kanaya AM, Chiang JL, Fujimoto W. BMI Cut Points to Identify At-Risk Asian Americans for Type 2 Diabetes Screening. Diabetes Care. 2015;38: 150–158. doi: 10.2337/dc14-2391 25538311 PMC4392932

[39] Misra A, Chowbey P, Makkar BM, Vikram NK, Wasir JS, Chadha D, et al. Consensus statement for diagnosis of obesity, abdominal obesity and the metabolic syndrome for Asian Indians and recommendations for physical activity, medical and surgical management. J Assoc Physicians India. 2009;57. 19582986

[40] Gupta R, Deedwania PC, Sharma K, Gupta A, Guptha S, Achari V, et al. Association of Educational, Occupational and Socioeconomic Status with Cardiovascular Risk Factors in Asian Indians: A Cross-Sectional Study. PLoS One. 2012;7. doi: 10.1371/journal.pone.0044098 22952886 PMC3430674

[41] Kinra S, Bowen LJ, Lyngdoh T, Prabhakaran D, Reddy KS, Ramakrishnan L, et al. Sociodemographic patterning of non-communicable disease risk factors in rural India: A cross sectional study. BMJ. 2010;341: 771. doi: 10.1136/bmj.c4974 20876148 PMC2946988

[42] Jaacks LM, Vandevijvere S, Pan A, McGowan CJ, Wallace C, Imamura F, et al. The obesity transition: stages of the global epidemic. Lancet Diabetes Endocrinol. 2019;7: 231–240. doi: 10.1016/S2213-8587(19)30026-9 30704950 PMC7360432

[43] LaVeist TA, Thorpe RJ, Galarraga JE, Bower KM, Gary-Webb TL. Environmental and socio-economic factors as contributors to racial disparities in diabetes prevalence. J Gen Intern Med. 2009;24: 1144–1148. doi: 10.1007/s11606-009-1085-7 19685264 PMC2762509

[44] Luo J, Rossouw J, Tong E, Giovino GA, Lee CC, Chen C, et al. Smoking and diabetes: does the increased risk ever go away? Am J Epidemiol. 2013;178: 937–945. doi: 10.1093/aje/kwt071 23817918 PMC3816526

[45] Foy CG, Bell RA, Farmer DF, Goff DC, Wagenknecht LE. Smoking and Incidence of Diabetes Among U.S. Adults. Diabetes Care. 2005;28: 2501–2507. Available: http://care.diabetesjournals.org/content/28/10/2501.abstract doi: 10.2337/diacare.28.10.2501 16186287

[46] Pan A, Wang Y, Talaei M, Hu FB, Wu T. Relation of active, passive, and quitting smoking with incident type 2 diabetes: A systematic review and meta-analysis. Lancet Diabetes Endocrinol. 2015;3: 958–967. doi: 10.1016/S2213-8587(15)00316-2 26388413 PMC4656094

[47] Saeed AA. Association of tobacco products use and diabetes mellitus-results of a national survey among adults in Saudi Arabia. Balkan Med J. 2012;29: 247–251. doi: 10.5152/balkanmedj.2012.035 25207009 PMC4115819

[48] Veldheer S, Yingst J, Zhu J, Foulds J. Ten-year weight gain in smokers who quit, smokers who continued smoking and never smokers in the United States, NHANES 2003–2012. Int J Obes. 2015;39: 1727–1732. doi: 10.1038/ijo.2015.127 26155918 PMC4976446

[49] Sahle BW, Chen W, Rawal LB, Renzaho AMN. Weight Gain after Smoking Cessation and Risk of Major Chronic Diseases and Mortality. JAMA Netw Open. 2021;10. doi: 10.1001/jamanetworkopen.2021.7044 33904915 PMC8080225

[50] Baliunas DO, Taylor BJ, Irving H, Roerecke M, Patra J, Mohapatra S, et al. Alcohol as a Risk Factor for Type 2 Diabetes. Diabetes Care. 2009;32: 2123–2132. doi: 10.2337/dc09-0227 19875607 PMC2768203

[51] Koloverou E, Panagiotakos DB, Pitsavos C, Chrysohoou C, Georgousopoulou EN, Metaxa V, et al. Effects of alcohol consumption and the metabolic syndrome on 10-year incidence of diabetes: The ATTICA study. Diabetes Metab. 2015;41: 152–159. doi: 10.1016/j.diabet.2014.06.003 25190450

[52] Carlsson S, Hammar N, Grill V, Kaprio J. Alcohol consumption and the incidence of type 2 diabetes: A 20-year follow-up of the Finnish Twin Cohort Study. Diabetes Care. 2003;26: 2785–2790. doi: 10.2337/diacare.26.10.2785 14514580

[53] Liu C, Yu Z, Li H, Wang J, Sun L, Qi Q, et al. Associations of alcohol consumption with diabetes mellitus and impaired fasting glycemia among middle-aged and elderly Chinese. BMC Public Health. 2010;10. 21092093 10.1186/1471-2458-10-713 PMC2998495

[54] He X, Rebholz CM, Daya N, Lazo M, Selvin E. Alcohol consumption and incident diabetes: The Atherosclerosis Risk in Communities (ARIC) study. Diabetologia. 2019;62: 770–778. doi: 10.1007/s00125-019-4833-1 30820594 PMC6451679

[55] Beulens JWJ, Van der Schouw YT, Bergmann MM, Rohrmann S, Schulze MB, Buijsse B, et al. Alcohol consumption and risk of type 2 diabetes in European men and women: Influence of beverage type and body size The EPIC-Inter Act study. J Intern Med. 2012;272: 358–370. doi: 10.1111/j.1365-2796.2012.02532.x 22353562

[56] Paulson QX, Hong J, Holcomb VB, Nunez NP. Effects of body weight and alcohol consumption on insulin sensitivity. Nutr J. 2010;9. 20307313 10.1186/1475-2891-9-14 PMC2859759

